# Self-preservation and Stability of Methane Hydrates in the Presence of NaCl

**DOI:** 10.1038/s41598-019-42336-1

**Published:** 2019-04-10

**Authors:** Pinnelli S. R. Prasad, Burla Sai Kiran

**Affiliations:** 1 Gas Hydrate Division, CSIR–National Geophysical Research Institute (CSIR–NGRI), Hyderabad, 500 007 India; 2 Academy of Scientific and Innovative Research (AcSIR), CSIR–NGRI Campus, Hyderabad, 500 007 India

## Abstract

Gas hydrate, a solid transformed from an ensemble of water and gaseous molecules under suitable thermodynamic conditions, is present in marine and permafrost strata. The ability of methane hydrates to exist outside of its standard stability zone is vital in many aspects, such as its utility in gas storage and transportation, hydrate-related climate changes and gas reservoirs on the planet. A systematic study on the stability of methane hydrates divulges that the gas uptake decreased by about 10% by increasing the NaCl content to 5.0 wt%. The hydrate formation kinetic is relatively slower in a system with higher NaCl. The self-preservation temperature window for hydrate systems with NaCl 1.5, 3.0 and 5.0 wt% dramatically shifted to a lower temperature (252 K), while it remained around 270 K for NaCl 0.0 and 0.5 wt%. Based on powder x-ray diffraction and micro-Raman spectroscopic studies, the presence of hydrohalite (NaCl·2H_2_O) phase was identified along with the usual hydrate and ice phases. The eutectic melting of this mixture is responsible for shifting the hydrate stability to 252 K. A systematic lattice expansion of cubic phase infers the interaction between NaCl and water molecules of hydrate cages.

## Introduction

The gas hydrates, a class of clathrate compounds, composed of several polyhedral cages made up of hydrogen-bonded water molecules. Those cages are filled with suitable guest molecules, such as methane, ethane, carbon dioxide, nitrogen, hydrogen sulfide. The gas hydrates look similar to ice, and the natural gas hydrates are also popularly known as “burning ice”. These materials are attractive for several reasons^1–6^. The volume of gas (guest) in a unit volume of hydrate is at least two orders of magnitude higher compared to the standard pressure (p) and temperature (T) conditions. The stability conditions (p and T) of hydrate phase are milder (compared to compressed form of natural gas -CNG or liquefied form of natural gas -LNG) and thus these solid gas hydrates (SGH), i.e., hydrates formed with natural gas as guests, are being considered as an economically feasible form for fuel gas storage and transportation. However, slow formation kinetics (several hours to days) and inefficient hydrate conversion are pertinent constraints for its large-scale applications. Usage of some porous materials as confining matrix for water molecules, and the addition of some surfactants/additives even in a smaller dosage (<1 wt%) were proven to be active promoters of gas hydrates, in particular, for methane hydrates^7–10^. Apart from gas storage applications, gas hydrate-based methodology has established its presence in the gas separation, water desalination, refrigeration technology and so on^11,12^.

The bulk of the experimental work on the phase and structural stability of methane hydrates focused on the binary system, i.e., H_2_O-CH_4_. Several experiments have also been conducted in this system with the aid of additives and porous sediments to understand the formation and dissociation kinetics^7–10^. The pioneering phase behaviour study of the ternary system, i.e., NaCl-H_2_O-CH_4_, is described by de Roo *et al*.^13^. Possible solid phases in this ternary system are NaCl·2H_2_O (hydrohalite), ice and hydrate, and these phases are in addition to the brine-liquid and gas phases. Investigations on these binary and ternary systems revealed that the melting of hydrohalite phase is independent of initial NaCl concentration in the solution and the gas pressure, whereas the ice melting is independent of gas pressure but strongly depend on NaCl concentration (vary along the brine-liquidus curve)^14–16^.

On the other hand, the hydrate phase melting depends strongly on both the gas pressure and NaCl content. In all these studies the NaCl concentration was rather high (>10 wt%). Earlier studies with higher salt content were exploited extensively to find the means of preventing hydrate formation in the process. Cha *et al*.^17^ have compared the phase equilibria of methane hydrates in the presence of NaCl, KCl and NH_4_Cl solutions, using the isochoric method and high-pressure differential scanning calorimetry (DSC) and inferred that the detected phase stability points from both the methods are consistent, and the hydrate inhibition is more for NaCl. More practical usage of salts in the gas hydrate related research is to use them as hydrate inhibitors in deep and ultradeep oil/gas transmission pipelines. Therefore, earlier gas hydrate studies were conducted at higher pressures (up to 200 MPa) and with higher salt concentration (up to 30 wt%)^18^. The results can be summarised as the following: (i) The phase equilibrium boundary moves towards lower temperature and higher pressure as the salt concentration increased to its saturation limit, and it remains unaltered even at higher concentrations. (ii) Hydrate formation is also possible with high salinity (above saturation limit) coupled with salt precipitation, and both of them are competing effects. (iii) Supersaturation of the solutions potentially self-inhibits hydrate formation due to strong electrostatic forces between ions and water, causing retardation in hydrate formation.

The stability of natural gas hydrate-bearing sediments is susceptible to fluctuations in the pressure/temperatures in their locations. Additionally, percolation of salt into the deposits also destabilises the gas hydrates by reducing the extension and the thickness of their thermodynamic stability zone. Recent studies by Riboulot *et al*.^19^ inferred that the fluctuations in the salinity also is a critical parameter controlling the stability of gas hydrates of the Black Sea, particularly at the near surface. You *et al*.^20^ studied the methane hydrate formation and dissociation with salinity-buffered solutions in the gas-rich region; and concluded that the salt is excluded during hydrate formation. Thus, changing the salinity of the solution causing a shift in the thermodynamic boundary.

Another unique property, associated with some gas hydrates, like CH_4_ and CO_2_, is commonly acknowledged as self- (or anomalous) preservation effect and which is useful in gas storage and transportation applications at ambient pressure^21,22^. According to this effect, the gas hydrates show abnormal stability even outside its thermodynamic stability limits and the gas encased in hydrates can be preserved for a longer duration. The rate of gas release (or hydrate dissociation) at atmospheric pressure exponentially increase with temperature, and the gas releasing rate significantly decreases at around 240 K and a similar trend will persist until 272 K. This temperature zone, i.e., 240 to 272 K is known as self-preservation zone. Although the precise mechanism for this unusual property is unknown, the formation of some ice layer around hydrate grains, an additional kinetic barrier for gas diffusion, is responsible. The self-preservation effect depends on several factors, such as guest gas composition, hydrate grain size, the presence of other additives and co-guest molecules, and so on^23^.

On the other hand, the role of electrolytes in hydrates, particularly in the vicinity self-preservation region is less known. Sato *et al*.^24,25^, have examined the decomposition rates of methane hydrate in the presence of dilute electrolyte solutions (≤34 mol/m^3^), in the temperature range 233 to 273 K, and reported that the decomposition is remarkably suppressed (slower than hydrates with pure water) immediately below the eutectic temperature. Authors have also observed a brief upsurge in the decomposition rate at the eutectic temperature. Mimachi *et al*.^26^, also have studied the dissociation behaviour of methane hydrates, prepared from 3.0 and 10 wt% NaCl solutions, and have reported a faster decomposition for hydrates synthesised with 10 wt% NaCl solutions at 253 K. While the decomposition behaviour for hydrate with 3.0 wt% NaCl solution is comparable to the pure water system.

The present study is aimed at revisiting the thermodynamic inhibiting nature of methane hydrates in the presence of NaCl, particularly in under-saturated condition. We also examined the self-preservation behaviour of methane hydrates synthesised with pure water (0.0 wt% NaCl), mild salt solutions (0.5 & 1.5 wt% NaCl) and salt content comparable with sea-water (3.0 & 5.0 wt%). Such studies will provide a detailed insight into the hydrate dissociation behaviour.

## Results and Discussion

### Hydrate formation in electrolyte solution

The methane hydrates were synthesised using an aqueous solution prepared from 0.0, 0.5, 1.5, 3.0 and 5.0 wt% NaCl. Additionally, 0.5 wt% l-methionine (l-met) was also added. Addition of l-met to the aqueous solution helps in efficient and rapid methane hydrate conversion even in non-stirred configuration^9^. We conducted all the experiments in constant volume mode by charging the reactor vessel with 29 mL of stock solution and pressurising it with ~7.5 to 8.0 MPa methane gas at ambient temperature (298 K). The schematic experimental set-up is shown in Supplementary Information Figure (SI-1).

An illustrative pressure-temperature (p-T) trajectory for each system is shown in Supplementary Figure (SI-2), wherein the black and red coloured dots denote recorded behaviour during cooling and thawing cycles respectively. The phase boundary curve is generated, using CSMGem model^1^ and is shown in the blue line. A sharp decrease in the methane gas pressure, coupled with a small temperature rise, indicative of state change to hydrate phase, is observed in all the systems. The completion/saturation of the phase changes are denoted by an insignificant pressure decrease over a longer time span at some lower temperature. The hydrates are dissociated in a thawing cycle by, increasing the temperature at a rate of 4–6 K/h. The fast heating can induce measurable drift from the computed phase boundary curve. However, it is desirable to conduct the dissociation at much slower viz 0.5 to 1.0 K/h ramping rate, to find the correct phase boundary point. However, the primary objective of the present work is not on the phase boundary points but to verify the gas uptake, kinetics and stability of methane hydrate system in the presence of NaCl. Thus, the hydrates were dissociated at a faster rate. We calculated the gas uptake/release during the hydrate formation/dissociation using Eq. 1, and the data from at least three repeated measurements were considered in all interpretations.

**Figure 1:**
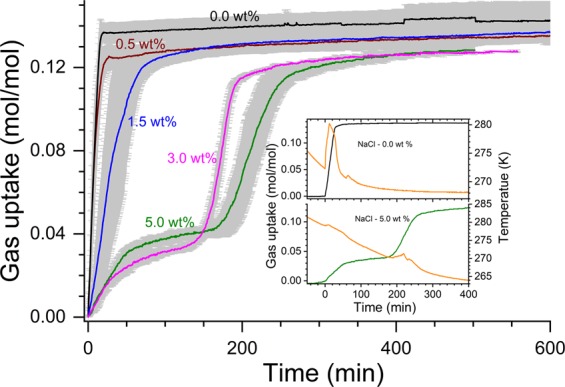
The methane gas uptake during hydrate formation in CH_4_-H_2_O-NaCl system in the first 600 min from the nucleation event. The dominant gas uptake for hydrate forming systems with 0.0, 0.5 and 1.5 wt% NaCl, while it occurred in two stages for 3.0 and 5.0 wt% NaCl. The inset shows recorded temporal variations in temperature (orange) and gas uptake for 0.0 and 5.0 wt% NaCl.

Figure 1 shows the average of gas uptake kinetics during the hydrate conversion process (first 600 min from the nucleation) in the presence of NaCl. The onset for hydrate nucleation is identified by an abrupt change in the pressure drop, and at the same instant, an exothermic temperature peak also appeared. The inset graphs show the temporal variations in the measured temperatures (hydrate/aqueous phase) for two systems (NaCl 0.0 & 5.0 wt%). The insets also depict the gas uptake kinetics for these two representative systems. As shown in the inset figures it is not always possible to have a stronger exothermic temperature peak. Several factors such as heat transfer efficiency, placement of sensor etc., will influence the detectability of the exothermic peak. Thus, the combination of an exothermic peak coupled with a change in the slope of vapour phase pressure is considered as a triggering point for the hydrate phase^27^.

**Figure 2:**
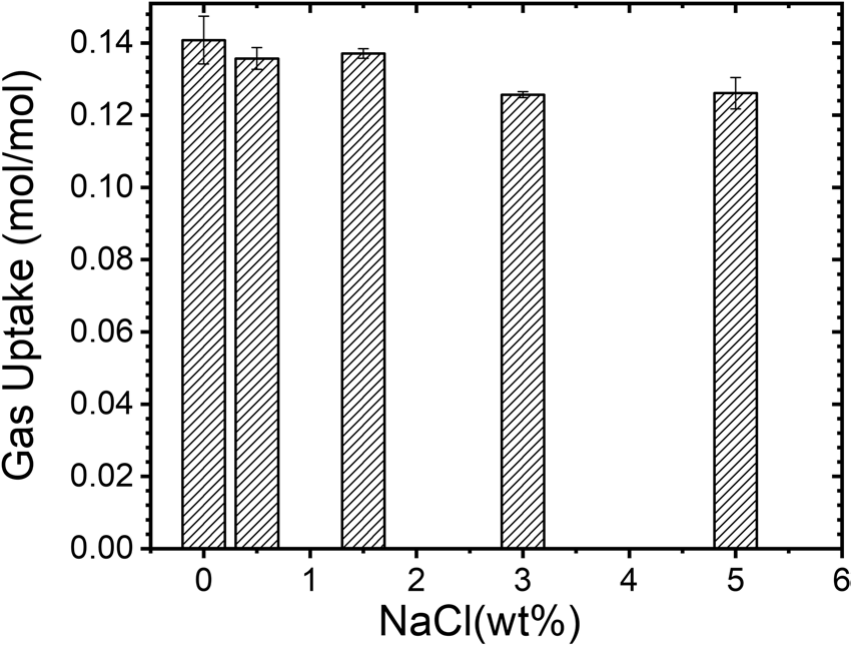
The gas uptake in the process of hydrate conversion in CH_4_-H_2_O-NaCl system.

Presence of electrolytes such as NaCl is a well-known thermodynamic inhibitor and also retards the gas uptake during the hydrate conversion. However, the time taken for 90% of hydrate conversion in no or less saline systems is really fast (i.e., 15 and 25 min for 0.0 and 0.5 wt% NaCl). On the other hand, the hydrate conversion process occurred in longer time spans for 1.5 (80 min), 3.0 (200 min) and 5.0 (260 min) wt% NaCl. The hydrate nucleation also occurred in multi-stages in higher (3.0 and 5.0 wt%) saline system. As said the electrolytes are thermodynamic inhibitors, while amino acid (l-met) is a good promotor for methane hydrates^9^. These contrary effects are responsible for the dual stage gas uptake, which has been observed predominantly in high saline systems. These results are in tandem with earlier reports. The overall gas consumption in hydrate formation, computed from p-T trajectories, (Fig. 2) shows a progressive decrement by about 10% at higher salinity. It is interesting to note that Chong *et al*.^28^, have reported similar observations in methane hydrates formed in the presence of NaCl (1.5 and 3.0 wt%) in a silica bed reactor. Those authors stated that the presence of NaCl induces a delay (1.5 times to pure water) in hydrate formation and also about 30% decrease in the hydrate conversion between NaCl solutions with 0.0 wt% and 3.0 wt%. Our results indicate that the addition of the amino acid is helpful for equivalent gas uptake in the pure and saline systems, leading to similar hydrate conversion.

### Self-preservation effect

**Figure 3:**
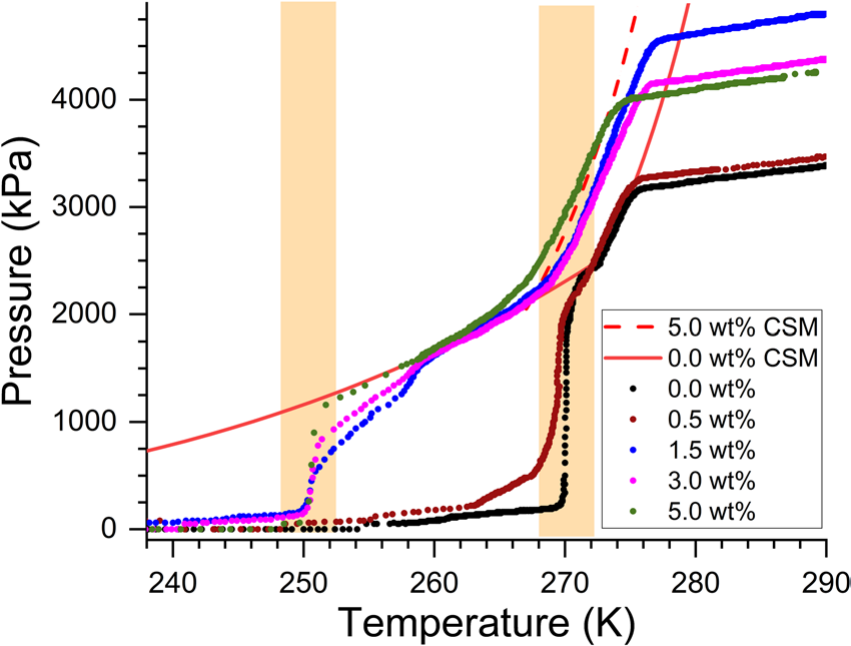
Observed methane pressure built-up during hydrate dissociation in CH_4_-H_2_O-NaCl system. The full and dash (red colour) lines indicate the computed phase boundary using CSMGem for 0.0 and 5.0 wt% NaCl systems.

Some gas hydrates are abnormally stable outside their general thermodynamic stability regions, and this property is valid only to some specific systems. The hydrate systems with guest molecules such as methane or carbon dioxide can be classified into this category. The mechanism for such a peculiar property is still not understood, but the hydrates are stable for more prolonged periods even at atmospheric pressures while preserving them at sub-zero temperatures. This property, readily identified as “self- or anomalous-“ preservation effect, has exciting applications in the gas storage & transportation sector^7,8^. In Fig. 3, we plot the dissociation behaviour of CH_4_ - H_2_O hydrate system in the presence of 0.0, 0.5, 1.5, 3.0 and 5.0 wt% NaCl, along with computed phase boundary curves of the end-membered (0.0 and 5.0 wt% NaCl) systems using CSMGem^1^. The reactor vessel was equilibrated to the atmospheric pressure by removing the residual methane gas at a lower temperature (~250 K), and the hydrate system firmly is in its metastable state. A small portion of hydrate sample is transferred to 8 mL pressure vessel (pre-cooled to 150 K) and subjected to dissociation by increasing the temperature (by placing it in a cotton filled glove box). The pressure build-up is remarkably sluggish in CH_4_ - H_2_O (0.0 wt% NaCl) system when the temperature is below 268 K. The gas increases rapidly in the temperature window of 268 to 271 K. At about 270.8 K the total pressure of the reactor reached to 2350 kPa, which is a phase boundary point for pure methane hydrates. After that, the gas release by hydrate dissociation is primarily driven by the thermodynamic conditions.

Similarly, the dissociation of hydrates, formed with 0.5 wt% NaCl, activated at a somewhat lower temperature. Further, increase in the salinity such as 1.5, 3.0 and 5.0 wt% brought some remarkable changes in the dissociation process (see Fig. 3), namely the quick release of methane gas observed in the temperature close to 252 K, and also the phase-boundary curve shifted to the inhibition (left) side. It is interesting to note that the rapid gas release occurred in two distinct temperature windows, such as 268 to 271 K and 249 to 253 K. The former temperature window is in the vicinity of ice melting point, while the latter corresponds to the eutectic temperature for hydrohalite (sodium chloride dihydrate: NaCl·2H_2_O) phase. A decrease in the freezing point of water with increasing NaCl content is a well-known phenomenon, and the growth of hydrohalite phase is probable in H_2_O-NaCl system at higher NaCl (~23 wt%) weight, otherwise brine ice will form at lower temperatures. However, according to Mimachi *et al*.^26^ the hydrate grains in the ternary system with 10 wt% NaCl, were surrounded by the rims of NaCl·2H_2_O and as such no such lamellar objects were reported at lower NaCl content.

### *Ex-Situ* Characterisation of Gas Hydrate

#### Powder xrd study

**Figure 4:**
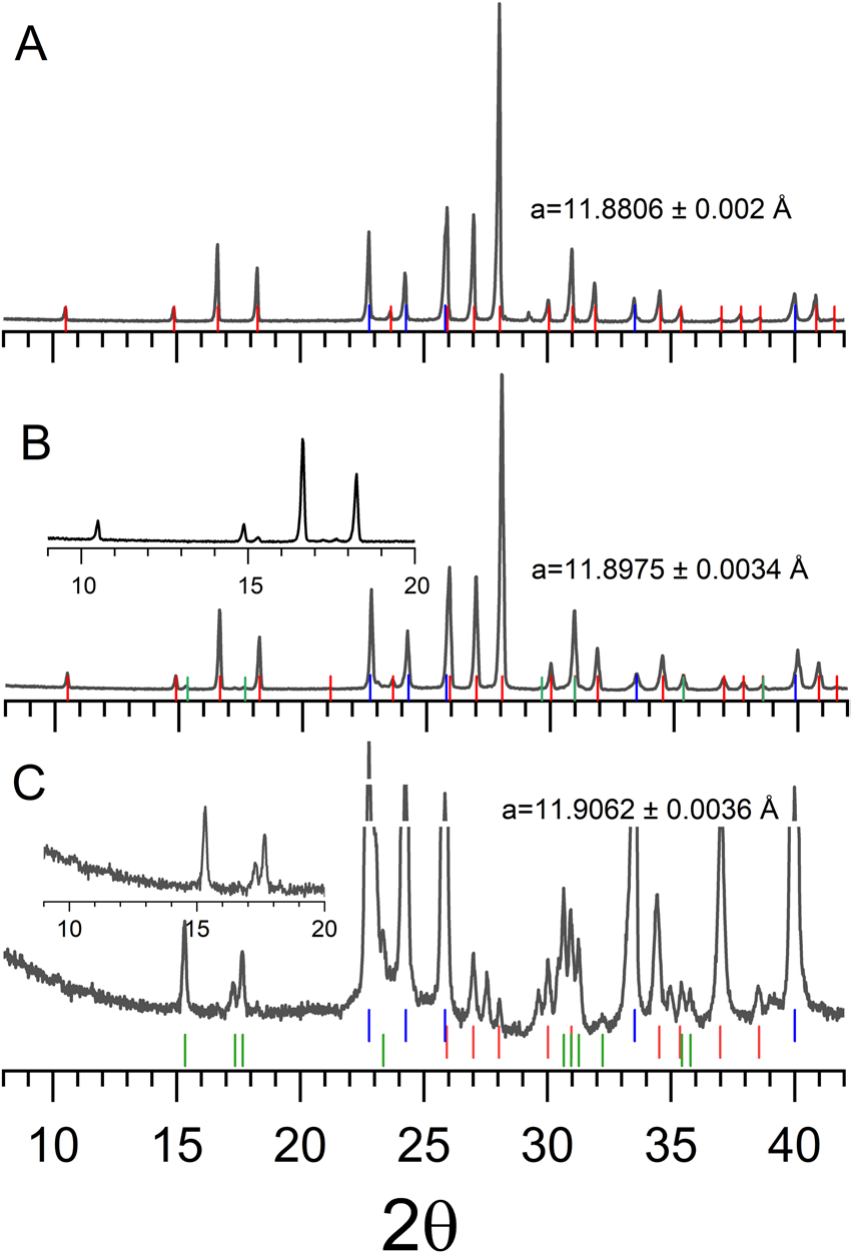
Recorded powdered X-ray diffraction patterns for the solid phases in the CH_4_-H_2_O-NaCl system. PXRD was recorded at 150 K. Top (**A**), and bottom (**C**) segments correspond to hydrate systems with 0.0, and 15.0 wt% NaCl and the middle (**B**) trace is with 3.0 wt% NaCl. Computed diffraction peak positions for cubic hydrate (Pm3n), hexagonal ice (P63/mmc) and monoclinic hydrohalite (P21/c) are represented by red, blue and green coloured bars respectively. Blown-up portions of the low-angle diffraction peaks are shown as insets for clarity.

Experimentally synthesised solid phases were characterised by the analytical techniques such as powder x-ray diffraction (PXRD) and laser Raman spectroscopy under ambient pressure and cryo-temperature conditions. The pressure vessel containing the hydrates were quenched to 150 K, by placing them in liquid nitrogen. The residual gas was completely removed intermittently, and the hydrate samples were preserved at low temperatures. Figure 4 shows the XRD pattern of hydrate samples recorded from fine powders (collected from different spatial locations of the hydrate sample) at 150 K with NaCl content 0.0, 3.0 and 15.0 wt%. Three solid phases possibly existed in H_2_O-NaCl-CH_4_ system, namely, methane hydrates (cubic), ice (hexagonal) and to some lesser extent hydrohalite (monoclinic). The recorded PXRD pattern was indexed by the CheckCell^29^ programme using space groups *Pm3n, P63*/*mmc and P21*/*c* respectively. The red, blue and green coloured bars indicate the computed positions for the cubic hydrate, hexagonal ice and hydrohalite phase respectively.

**Figure 5:**
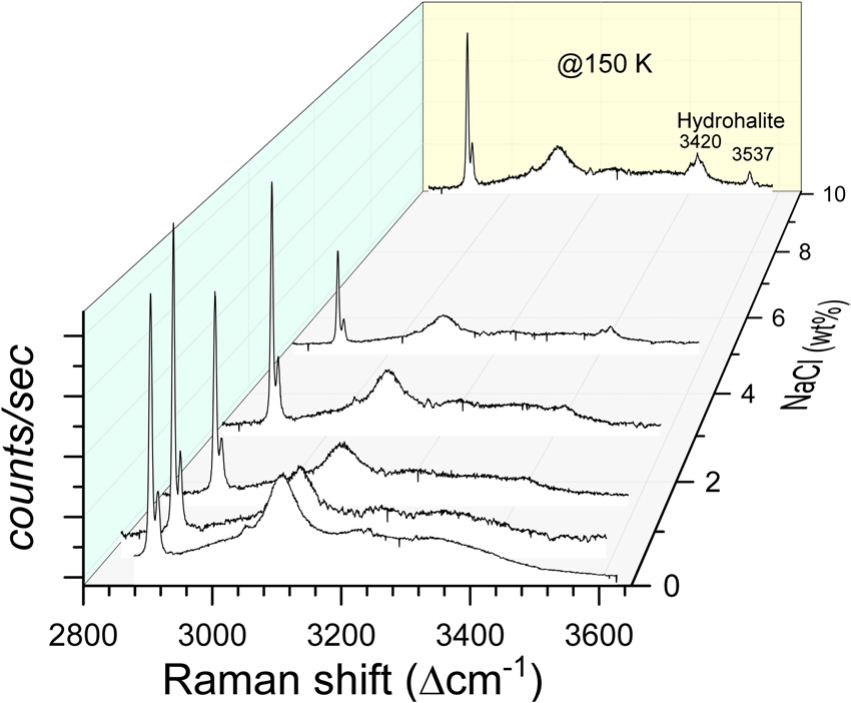
Recorded Raman spectra of hydrates in the wavenumber window 2800–3650 cm^−1^ for CH_4_-H_2_O-NaCl system. All the spectra were recorded at 150 K.

Observed PXRD pattern for the hydrate-forming system without NaCl (see Fig. 4A) is dominant of cubic hydrate phase features, and the unit cell length is estimated as 11.8806 ± 0.002 Å. In particular, the observed pattern around low-angles (2θ) consisting of 10.522 (*0 1 1*); 14.902 (*0 0 2*); 16.676 (*1 0 2*) and 18.282 (*1 1 2*) indicate the presence of hydrate phase (*Pm3n*). The hexagonal ice phase (*P63/mmc*) has distinguishable diffraction peaks at 22.796 (*0 1 0*); 24.265 (*0 0 2*); 25.954 (*0 1 1*) and 33.531 (0 *1 2*). On the other hand, the hydrate-forming system with NaCl as 15 wt% (synthesised to collect the specific signatures of the monoclinic phase) show a distinctly different pattern (inset of Fig. 4C). Three low-angle peaks are observed at 15.335 (*1 0 0*), 17.506 (*0 2 0*) and 17.673 (−*1 1 0*). These characteristic features of hydrohalite phase are distinctly different from the cubic hydrate phase, and they are absent. However, as shown in Fig. 5, the characteristic Raman features for CH_4_ molecules encased in hydrate cages are visible. Thus, the hydrate phase and the hexagonal ice also coexisted along with hydrohalite. As shown in Fig. 4C the PXRD lines for hydrohalite and hydrate peaks are weaker than the ice phase. The lattice parameter of the hydrate phase is estimated as 11.9062 ± 0.0036 Å. The hydrate-forming system with 3.0 wt% NaCl (Fig. 4B) shows mixed features, namely, dominant hydrate and weaker ice and hydrohalite signatures. Presence of hydrate and hydrohalite phases are observed from the characteristic low-angle peaks (see inset). The lattice parameter for this system is estimated as 11.8975 ± 0.0034 Å. Similar XRD pattern namely the co-existence of hydrate and hydrohalite phases are observed in hydrate forming systems with NaCl. The PXRD patterns for other NaCl (0.5, 1.5 and 6.0 wt%) concentrations are shown in the Supplementary Information (SI-3).

#### Micro–raman spectroscopic study

A small portion of hydrate sample (selected from different spatial locations) is placed on the LINKAM FTIR-600 stage, which was pre-cooled to 150 K. Spectral window in the range 2800 to 3700 cm^−1^ will have the characteristic signatures for methane molecules encased in the hydrate phase and also the OH stretching mode of network water molecules. Further, the typical modes for the hydrohalite phase are also seen prominently in this spectral window. In Fig. 5, we show the Raman spectrum for methane hydrates prepared in the presence of NaCl. The characteristic vibrational bands of methane molecules encased in 5^12^6^2^, and 5^12^ cages of sI are observed at 2905 and 2915 cm^−1^ ^9,30^. Also, a broader band around 3106 cm^−1^ is a signature band for OH stretching mode for sI^31^. It is interesting to note some additional bands in this window, such as a triplet at 3406, 3420 and 3436 cm^−1^ and another band at 3537 cm^−1^ ^32,33^, particularly in the hydrate-forming system with 10 and 15 wt% NaCl. These bands also observed with lesser intensity for the systems with NaCl 1.5, 3.0, 5.0 wt%. Interestingly the hydrohalite signatures always appeared along with gas hydrate signatures and thus inferred that the hydrate grains are enclosed by a layer of hydrohalite. Further, such characteristic modes of hydrohalite phase are invisible in the hydrates synthesised with 0.0 and 0.5 wt% NaCl. Thus, the additional features appearing in the spectral window, particularly at higher NaCl content, indicates the presence of hydrohalite phase along with methane hydrates and ice phases. These observed features are grossly independent of the spatial location of the test specimen indicating the steady growth of hydrate. Our PXRD and Raman spectroscopic analysis explicitly indicate the presence of three solid phases namely cubic hydrate, hexagonal ice and monoclinic hydrohalite. The hydrohalite phase is particularly abundant at higher NaCl content.

#### Temperature dependent micro–raman spectroscopic study

**Figure 6:**
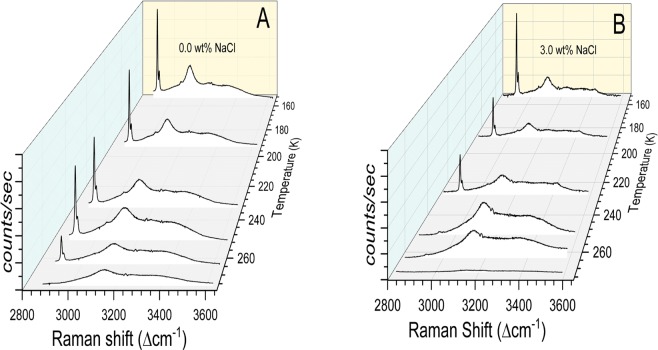
The Raman spectrum of hydrates samples synthesised with 0.0 wt% (**A**) and 3.0 wt% (**B**) NaCl at different temperatures.

**Figure 7:**
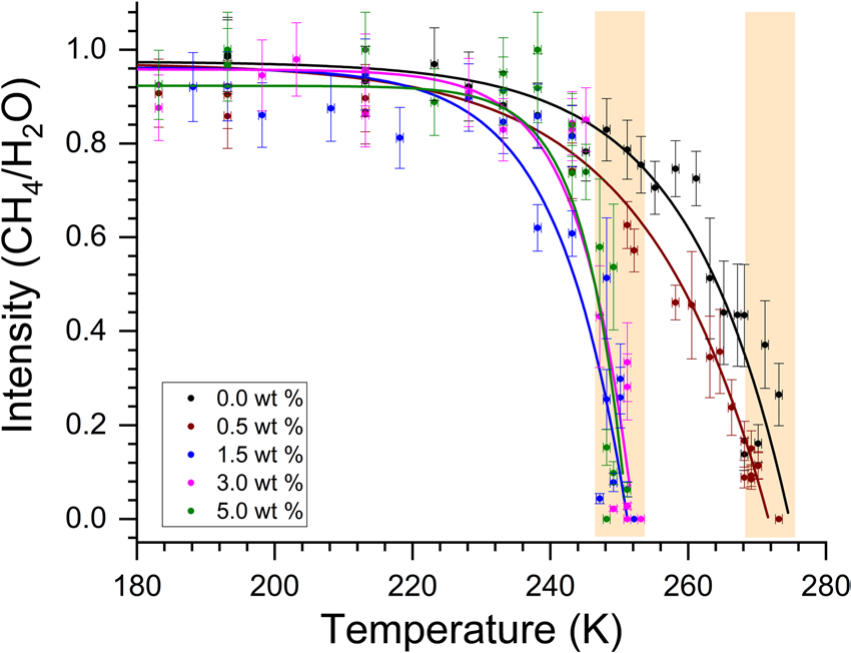
The variation in the amount of trapped CH_4_, measured from the intensity ratio the characteristic stretching modes of encased CH_4_ and cage-network H_2_O molecules, at different temperatures. The shaded vertical bars (guide to the eye only) show significant gas release in two different temperature windows. All the experiments were conducted at ambient pressure (@ 101 kPa).

To elucidate the thermal stability of CH_4_ molecules entrapped in the hydrate cages at ambient pressure, we carried-out detailed Raman spectroscopic studies in the range 150–273 K. The Raman spectrum of methane hydrates with 0.0 and 3.0 NaCl wt% at different temperatures is shown in Fig. 6. The samples are exposed to ambient pressure conditions. Similarly, the thermal evolution for hydrate systems with NaCl wt% (0.5, 1.5, 5.0) is shown in the Supplementary Information (as SI-4). The Raman signatures of methane molecules in hydrates without NaCl, namely, the bands at 2905 and 2915 cm^−1^ diminish as the temperature approaching ice melting point, while those with 3.0 wt% NaCl decrease close to 252 K. This behaviour is similar to the degassing observed in the pressure vessels (see Fig. 3). Variation in the amount of methane encased in the hydrate cages (measured as the ratio for the characteristic CH_4_ band to the H_2_O band at 3106 cm^−1^) by increasing the temperature is shown in Fig. 7. Interestingly, the ratio rapidly decreased around 273 K (ice melting temperature) for hydrated synthesised with 0.0 and 0.5 wt% NaCl, while the ratio dramatically reduced around 252 K for all other systems with higher NaCl content. Therefore, the hydrates with a certain amount of NaCl are less stable.

#### Mechanism of self-preservation

Although the self- (or anomalous) preservation effect of methane hydrate is a well-known phenomenon, the exact molecular mechanism is still puzzling. Nevertheless, this property is very much useful for gas transportation applications. The requirement of high-pressure vessels could be avoided as the hydrates show more extended stability in its self-preservation window^21–23,34^. However, previous studies suggest the following: the gas from gas hydrates diffuses rapidly in the temperature less than 240 K, and a thin water layer (product in the dissociation process) transforms into ice particulates and forms layer around hydrate grains. This layer of ice acts as an additional barrier for gas diffusion and prevent hydrate dissociation. Thus, the hydrates continue to stay in the meta-stable conditions until the temperature reaches the ice melting point.

As seen from the data plotted in Figs 3 and 7 the degassing of methane hydrates formed in the presence of NaCl occurred in two different temperature windows. The meta-stability of hydrates with pure and mild (0.5 wt%) NaCl solutions persists until ice melting temperature. The hydrates formed using a higher amount of NaCl have shown rapid gas release around 252 K. Interestingly, this is the eutectic temperature for the hydrohalite phase formed in NaCl-H_2_O system. The NaCl·2H_2_O usually develop from the solution at higher NaCl wt%. However, the present PXRD and Raman spectroscopic results (Figs 4 and 5) indicate the existence of this hydrohalite phase even with lower NaCl wt%. Further, a significant downshift of the meta-stable region to the eutectic temperature suggests that the hydrohalite may exist as defects in the protective ice layer around the hydrate grains. Thus, rapid gas diffusion is possible around the eutectic temperature. As said, the formation of hydrohalite requires saturated NaCl solutions, and this could have been facilitated by the accumulation of excess NaCl during the hydrate formation. Significant rejection of dissolved salts during the hydrate growth process is a well-known phenomenon, and as such, the gas-hydrate based methodology is useful in the desalination process.

**Figure 8:**
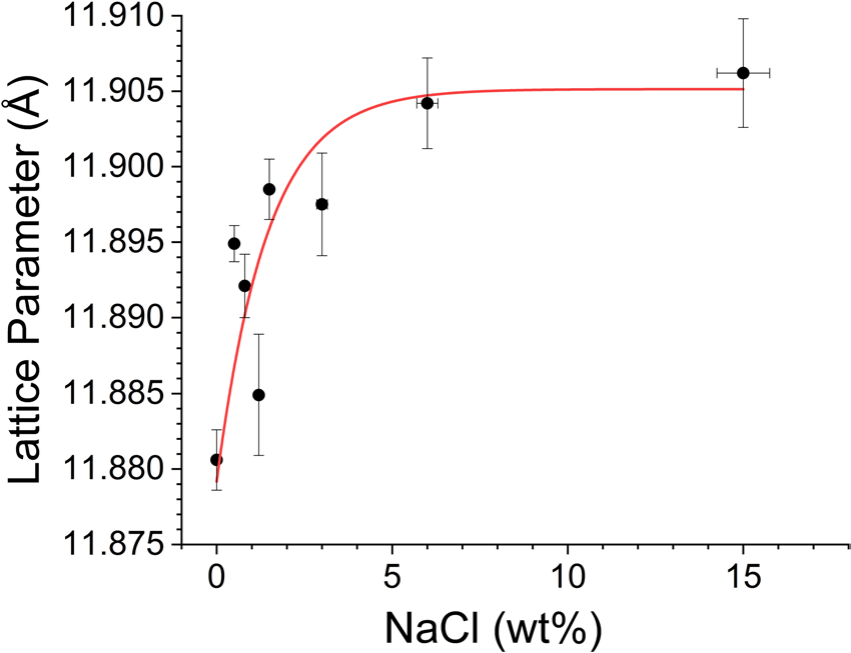
Estimated lattice parameter of the hydrate phase with different NaCl content. The red coloured line is a guide to the eye.

The lattice parameter of the cubic hydrate phase also increases by increasing the concentration of NaCl. These variations are systematically plotted in Fig. 8. The line is a guide to the eye. Estimated lattice parameter for the pure system is 11.8806 Å, and it increases to 11.906 Å for hydrates with NaCl 6.0 wt%, and it remained the same for 15 wt% NaCl system. The extent of change is small (0.22%) but systematic. This indicates an interaction between Na^+^ with the H_2_O molecules of hydrate cages. Cha *et al*.^17^, have shown that the hydrates with NaCl are better inhibitors than KCl and NH_4_Cl and this is because of their ability to interact more with the water molecules in the hydration layer. Further, Sa *et al*.^35^, have also shown that the amino acid molecules are incorporated into the hydrate cages leading to expansion of the lattice as a function of amino acid content.

An interesting point to note is the dissociation trend at somewhat high pressures (>2000 kPa) for hydrates (see Fig. 3). The dissociation trend strictly follows the thermodynamic phase boundary curve for pure hydrates. On the other hand, the dissociation pattern for the hydrates prepared with NaCl 1.5, 3.0 and 5.0 wt% is shifted to inhibition side and follows the thermodynamic phase boundary for NaCl system. It is worthwhile to recall that the stability of methane hydrates in the self-preservation window depends on several factors and granular size is one among them. Nakoryakov and Misyura^34^ have shown that the gas diffusion in natural hydrates (0.7 mm) is slower than the synthetic hydrates (2.4 mm), although the average granular size of natural hydrates is considerably less. On the other hand, Falenty *et al*.^23^, have reported that the gas diffusion from unconsolidated hydrate grains, synthesised from 0.3 mm or less, ice particles is significantly faster compared to highly consolidated hydrate grains. Thus, the stability of gas hydrates in its meta-stable window is more complicated and the properties of the solid ice layer, which is acting as an additional barrier for gas diffusion, also attains considerable significance. It is essential to have perfect packing, and thicker ice crust around hydrate grains, and also lesser defects will ensure greater hydrate stability even in the meta-stable region.

As said, the phase boundary points for the ternary system such as CH_4_-H_2_O-NaCl has been experimentally verified at higher pressures, and it is not possible to compute the same from CSMGem^1^ also at low pressures and temperature conditions. Extrapolation of the phase boundary using some proposed empirical relations^13,16^ from the literature causes significant deviations from the CSMGem model. It is difficult to understand the self-preservation effect as these empirical relations predicts the hydrate stability at significantly lesser pressures than CSMGem model (see SI-5). Nevertheless, the salt-solutions are well-known thermodynamic inhibitors for methane hydrates, and thus it is fair to assume that the stability pressure is more than that of pure hydrates at a given temperature. In other words, we consider the phase boundary for hydrates synthesised with and without NaCl to be identical at lower temperatures. As shown in Fig. 3 (Fig. also SI-5) the dissociation of hydrates, prepared with the lesser amount of NaCl, is predominately around ice/brine -melting temperature. There is a significant reduction in the gas release rate upon cumulative pressure approaching equilibrium pressure (at a given temperature). Plausibly of a sudden upsurge in the gas release around 252 K, in the hydrates with a higher amount of NaCl, is because of the eutectic melting of hydrohalite defects in the brine-ice layer surrounding hydrate grains. Both the PXRD and Raman spectroscopic investigations show the presence of hydrohalite and also its abundance increases with NaCl content.

### Conclusions

In summary, we systematically examined methane hydrate formation behaviour of the aqueous solution with (0.0 to 5.0 wt%) weight fraction of NaCl, under isochoric and non-stirred configuration. The methodology could be easily adaptable for large-scale applications. Overall water to hydrate conversion is 88.68% for 0.0 wt% NaCl system, while it decreased progressively to 79.24% in 5.0 wt% NaCl system. Transformation into the hydrate phase is rapid and occurred in a single step in lower NaCl (1.5 wt%) contents, while it is considerably slower and happened in two stages in hydrate forming systems with higher NaCl. By PXRD and micro-Raman investigations, co-existence of hydrohalite phase, particularly in the systems with higher NaCl contents, is established. The significantly rapid gas release is detected around 252 K, which is a eutectic temperature for brine-ice and hydrohalite, for the methane hydrates formed with 1.5, 3.0 and 5.0 wt% NaCl. Whereas, the methane hydrates from 0.0 and 0.5 wt% NaCl are stable up to ice-melting temperature. Thus, self-preservation of the methane hydrates is strongly influenced by the presence of NaCl.

## Experimental Section

The de-ionized ultra-pure water (Millipore–type 1) was used, and dissolved gases were removed by evacuation. High purity (99.95%) methane was acquired from M/S Bhuruka Gas Company.

### Apparatus

The hydrate crystalliser, the central part of the experimental setup, is made up of a solid SS-316 rod, which can withstand gas pressures up to 20 MPa, and volume of the vessel was 250 mL. The inside temperature of the hydrate crystalliser was maintained at a desired level by immersing it in a cold fluid (water + glycol mixture) tank, coupled with a closed loop chiller. A platinum resistance thermometer (Pt100) was inserted into the vessel to measure the temperature of aqueous/hydrate, with an accuracy of ±0.5 K. The pressure in the vessel was measured with the pressure transducer (WIKA, type A-10 for pressure range 0–25 MPa with ±0.5% accuracy). The small vessel (8.0 mL), used for gas release measurements, has a similar arrangement for p &T logging.

### Procedure

The experiments were conducted in constant volume mode. Initially required amount (29 g) aqueous solution was added to the hydrate crystalliser and was pressurised with experimental values using Teledyne ISCO syringe pump. The atmospheric gas was removed by purging three/four times with methane gas (1.0 MPa). The reactor vessel was completely isolated from the gas tank assembly and was immersed in the tank with a cold fluid to lower the temperature. At some suitable temperature, a sharp decrease in the gas pressure indicates the state change. The irrelevant head-pressure drop in the crystalliser over a longer duration specifies the saturation in hydrate conversion. The temperature and pressure were logged for every 30 seconds interval. The molar concentration of methane gas (Δ*n*H, *t*) in the hydrate phase at time *t*, is defined by the following equation:

1 $${\rm{\Delta }}n{\rm{H}},t=ng,0-ng,t=({P}_{0}V/{Z}_{0}{{\rm{RT}}}_{0})-({P}_{t}V/{Z}_{t}{{\rm{RT}}}_{t})$$

The compressibility factor (*Z*) is calculated by the Peng-Robinson equation of state. The gas volume (*V*) was presumed as constant during the experiments, i.e., the volume changes due to phase transitions were neglected. *n*g, 0 and *n*g, *t* represent the number of moles of feed (methane) gas at zero time and in the gas phase at time *t*, respectively.

### Raman measurements

The Raman spectroscopic measurements were conducted on Horiba-T64000 system, coupled with an air-cooled argon ion laser emitting 514.5 nm which is the excitation source. We used LINKAM FTIR 600 cryo-stage is used to collect the Raman spectra at different temperatures in the range 153–300 K. The samples were exposed to laser radiation, using a 50X long distance focusing lens, only during the data accumulation. The Raman data was processed using GRAMS/3 software, and overlapped Raman bands were fitted into several Lorentzian components. The peak position, width and intensity for all the constituents were allowed to vary as free parameters for a convergent fitting.

### Powder x-ray diffraction measurements

The crystalline phases were analysed on a Bruker (Advance D8) diffractometer. The diffractometer was operational in the θ/2θ scan mode and the x-rays were having the wavelength of 1.5406 Å (Cu- radiation). An Anton-Paar (TTK-450) non-ambient stage was fixed in the sample chamber, and all the measurements were conducted at 150 K. The PXRD pattern was recorded in the range 2θ = 8 to 60°, in step scan mode. The dwell time and step size respectively are 0.5 sec and 0.02°.

## Supplementary information

Self-preservation and Stability of Methane Hydrates in the Presence of NaCl

## References

[1] Sloan, E. D. & Koh, C. A. Clathrate hydrates of natural gases, 3^rd^ ed., CRC Press, Boca Raton, F.l., (2008).

[2] Koh CA, Sloan ED, Sum AK, Wu DT (2011). Fundamentals and applications of gas hydrates. Annu. Rev. Chem. Biomol. Eng..

[3] Uchida T (2017). Review of fundamental properties of gas hydrates: breakout sessions of the international workshop on methane hydrate research and development. Energies.

[4] Sloan ED (2003). Fundamental principles and applications of natural gas hydrates. Nature.,.

[5] Chong ZR, Yang SHB, Babu P, Linga P, Li X-S (2016). Review of natural gas hydrates as an energy resource: Prospects and challenges. Applied Energy.

[6] Ruppel CD, Kessler JD (2017). The interaction of climate change and methane hydrates. Rev. Geophys..

[7] Borchardt L, Casco ME, Silvestre-Albero J (2018). Methane hydrate in confined spaces: an alternative storage system. Chem Phys Chem.

[8] Prasad PSR (2015). Methane hydrate formation and dissociation in the presence of hollow silica. J Chem Eng Data..

[9] Prasad PSR, Kiran BS (2018). Clathrate hydrates of greenhouse gases in the presence of natural amino acids: storage, transportation and separation applications. Scientific Reports.

[10] Cha M (2013). Thermodynamic and kinetic hydrate inhibition performance of aqueous ethylene glycol solutions for natural gas. Chem. Eng. Sci..

[11] Sun Q, Kang YT (2016). Review on CO_2_ hydrate formation/dissociation and its cold energy application. Renewable and Sustainable Energy Reviews.

[12] Dashti H, Yew LZ, Lou X (2017). Recent advances in gas hydrate-based CO_2_ capture. J Nat Gas Sci Eng..

[13] De Roo JL, Peters CJ, Lichtenthaler RN, Diepen GAM (1983). Occurrence of methane hydrate in saturated and unsaturated solutions of sodium chloride and water in dependence of temperature and pressure. AIChE Journal.

[14] Koop T, Kapilashrami A, Molina LA, Molina MJ (2000). Phase transitions of sea-salt/water mixtures at low temperatures: implications for ozone chemistry in the polar marine boundary layer. J Geophysical Res..

[15] Kharrat M, Dalmazzone D (2003). Experimental determination of solubility conditions of methane hydrate in aqueous calcium chloride solutions using high pressure differential scanning calorimetery. J. Chem. Thermodynamics.

[16] Maekawa T, Itoh S, Sakata S, Igari S, Imai N (1995). Pressure and temperature conditions for methane hydrate dissociation in sodium chloride solutions. Geochem J..

[17] Cha M, Hu Y, Sum AK (2016). Methane hydrate phase equilibria for systems containing NaCl, KCl and NH_4_Cl. Fluid Phase Equilibria.

[18] Hu Y (2017). Gas hydrates phase equilibria and formation from high concentration NaCl brines up to 200 MPa. J Chem Eng Data.

[19] Riboulot V (2018). Freshwater lake to salt-water sea causing widespread hydrate dissociation in the black sea. Nature Communications.

[20] You K, Kneafsey TJ, Flemings PB, Polito P, Bryant SL (2015). Salinity-buffered methane hydrate formation and dissociation in gas-rich systems. J. Geophy Res Solid Earth.

[21] Takeya S, Ripmeester JA (2008). Dissociation behavior of clathrate hydrates to ice and dependence on guest molecules. Angew. Chem. Int. Ed..

[22] Prasad PSR, Chari VD (2015). Preservation of methane gas in the form of hydrates: Use of mixed hydrates. J Nat Gas Sci Eng.

[23] Falenty A, Khus WF, Glockzin M, Rehder G (2014). Self-preservation of CH4 hydrates for gas transport technology: pressure-temperature dependence and ice microstructures. Energy Fuels.

[24] Sato H (2013). Self-preservation of methane hydrate revealed immediately below the eutectic temperature of the mother electrolyte solution. Chem Eng Sci..

[25] Sato, H. *et al*. Preservation of methane hydrates prepared from dilute electrolyte solutions. *Int J Chem Eng*. 843274, 10.1155/2009/843274 (2009).

[26] Mimachi H (2016). Dissociation behavior of methane hydrates formed from NaCl solutions. Fluid Phase Equilibria.

[27] Sowjanya K, Prasad PSR (2016). Sustainability of hollow silica matrix for clathrate hydrate recycling. J Nat Gas Sci Eng..

[28] Chong ZR, Chan AHM, Babu P, Yang M, Linga P (2015). Effect of NaCl on methane hydrate formation and dissociation in porous media. J Nat Gas Sci Eng..

[29] The software programme “Checkcell” developed by Laugier, L., Bochu, B. Laboratoire des Materiaux et du Genie Physique, Ecole Superieure de Physique de Grenoble is available at, http://www.ccp14.ac.uk.

[30] Chari VD, Prasad PSR, Murthy SR (2014). Structural stability of methane hydrates in porous medium: Raman spectroscopic study. Spectrochimica Acta Part A: Molecular and Biomolecular Spectroscopy.

[31] Schicks JM, Erzinger J, Ziemann MA (2005). Raman spectra of gas hydrates – differences and analogies to ice Ih and (gas saturated). water, Spectrochimica Acta Part A: Molecular and Biomolecular Spectroscopy.

[32] Ni P, Ding J, Rao B (2006). *In situ* cryogenic Raman spectroscopic studies on the synthetic fluid inclusions in the systems H_2_O and NaCl-H_2_O. Chinese Science Bulletin.

[33] Berg, R. W. Raman detection of hydrohalite formation: avoiding accidents on icy roads by deicing where salt will not work, *Applied Spectroscopy Reviews*, 10.1080/05704928.2017.1396540 (2017).

[34] Nakoryakov VE, Misyura SY (2013). The features of self-preservation for hydrate systems with methane. Chem Eng Sci..

[35] Sa J-H, Kwak G-H, Lee BR, Ahn D, Lee K-H (2014). Abnormal incorporation of amino acids into the gas hydrate crystal lattice. Phys. Chem. Chem. Phys..

