# Supplementary material for: Self-preservation and Stability of Methane Hydrates in the Presence of NaCl
Source: Sci Rep. 2019 Apr 10;9:5860. doi: 10.1038/s41598-019-42336-1 (PMC6458167; doi:10.1038/s41598-019-42336-1)
Supplement: Supplementary file 1 — Self-preservation and Stability of Methane Hydrates in the Presence of NaCl [file 41598_2019_42336_MOESM1_ESM.pdf]

<u>*Supplementary Information*</u>

Self-preservation and Stability of Methane Hydrates in the Presence of NaCl

PINNELLI. S.R. Prasad,[1,*] and BURLA. Sai Kiran[1,2]

[1]Gas Hydrate Division, CSIR–National Geophysical Research Institute (CSIR–NGRI)

[2]Academy of Scientific and Innovative Research (AcSIR), CSIR–NGRI Campus,

HYDERABAD – 500 007 (India)

Corresponding author's e-mail: psrprasad@ngri.res.in; Phone: +91 40 2701 2710;

Fax: +91 40 2717 1564

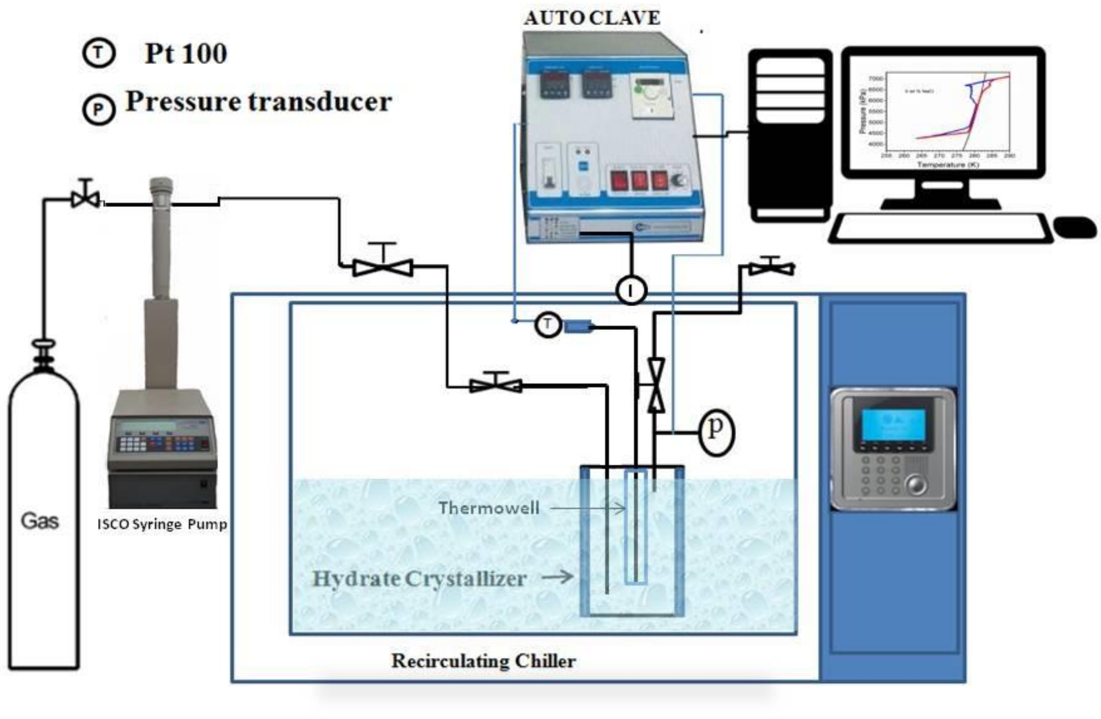

Figure SI-1: The schematic layout of the experimental set-up

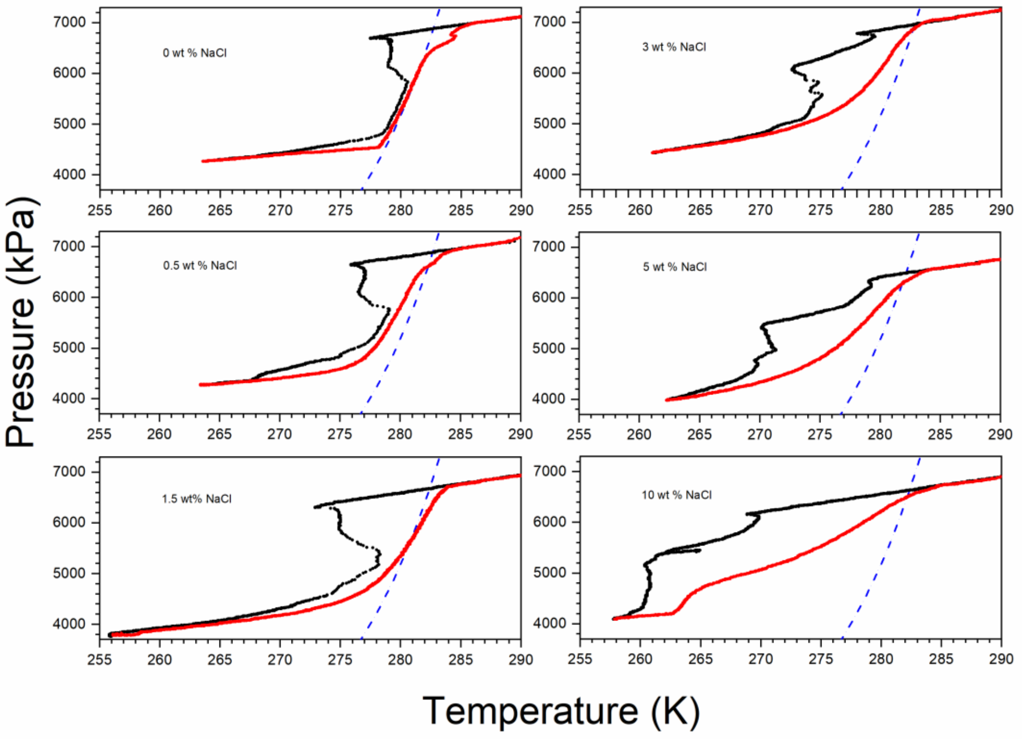

Figure SI-2: Recorded pressure (p) and temperature (T) trajectories depicting hydrate formation (black) and dissociation (red) process in CH_4 – H_2O – NaCl system. The blue coloured line shows the phase boundary curve of pure methane hydrates computed using CSMGem model.

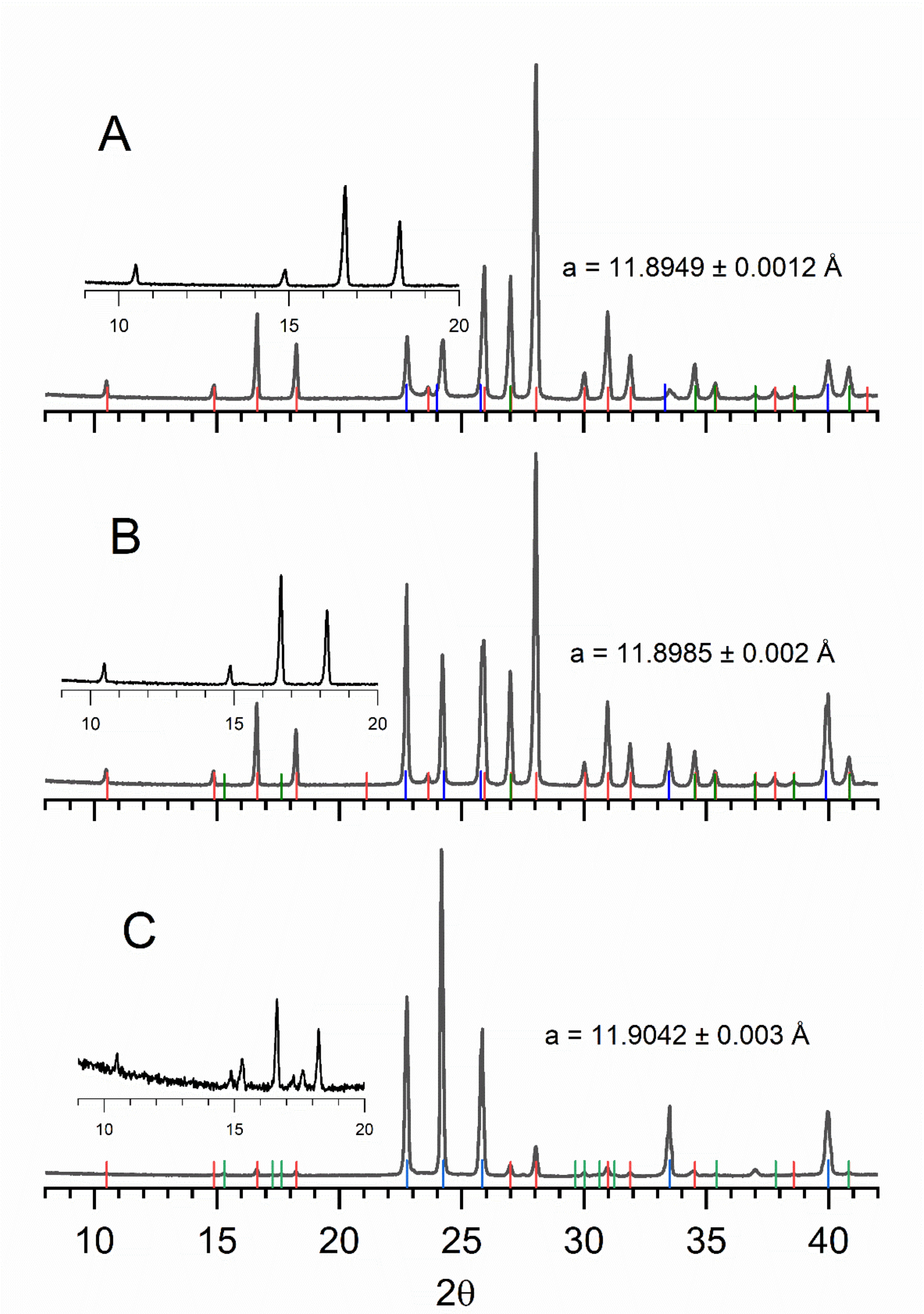

Figure SI-3: Recorded powdered X-ray diffraction patterns for the solid phases in the CH_4 – H_2O – NaCl system. PXRD was recorded at 150 K. Traces A, B & C correspond to hydrate systems with 0.5, 1.5, and 6.0 wt% NaCl. Computed diffraction peak positions for cubic hydrate (Pm3n), hexagonal ice (P63/mmc) and monoclinic hydrohalite (P21/c) are represented by red, blue and green coloured bars respectively. Blown-up portions of the low-angle diffraction peaks are shown as insets for clarity.

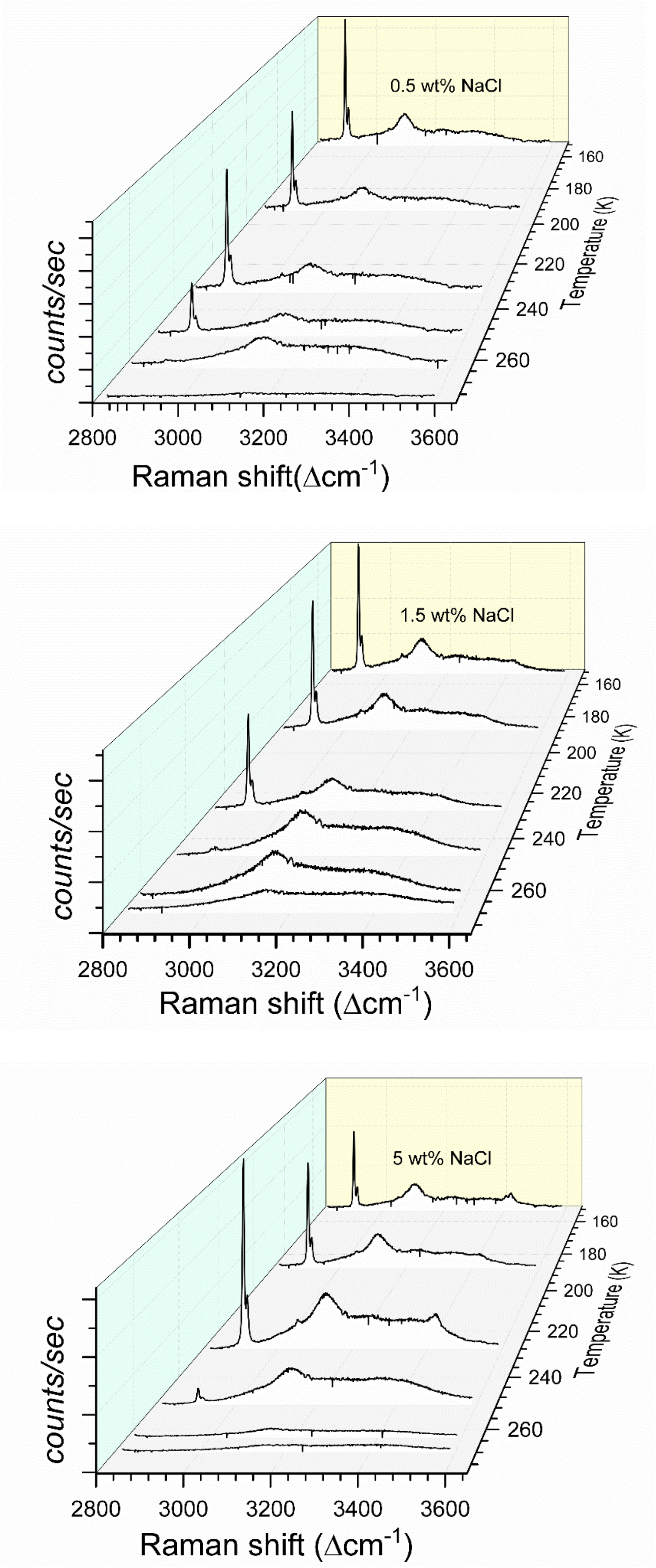

Figure SI-4: Recorded Raman spectrum of methane hydrates at different temperatures.

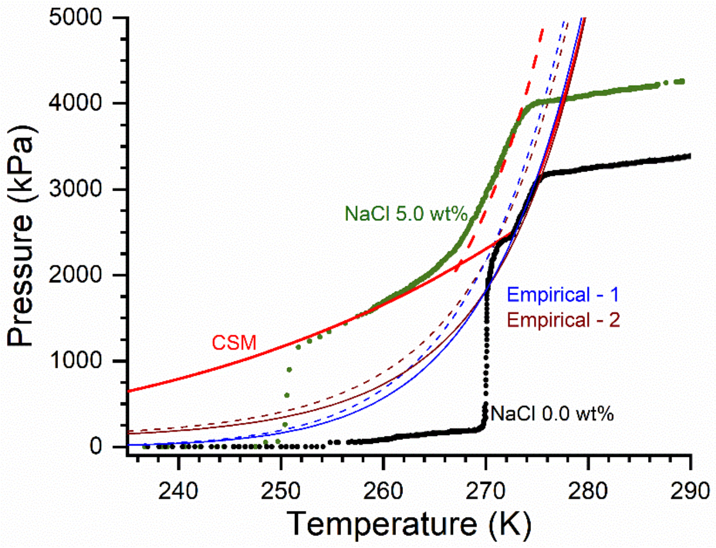

Figure SI-5: The dissociation pattern of hydrates synthesised with 5.0 wt% (green dots) and 0.0 wt% (black dots). Computed phase boundary curves using CSMGem (red) model and extrapolated traces using the empirical relations proposed in reference #13 (blue) and #16 (brown) for comparison are also shown. The dashed and solid computed lines respectively are for 5.0 and 0.0 wt% NaCl systems.
